# Professional Sentiment as Evidenced by the Medical Press

**Published:** 1885-10

**Authors:** 


### PROFESSIONAL SENTIMENT AS EVIDENCED BY THE MEDICAL PRESS.

The *New York Medical Record*, first fiddler, and the *Columbus Medical Journal*, second ditto and "Me Too" to the Obstructionists' New Code Can-can, grossly misrepresent the state of public sentiment as voiced by the medical press. The former declares that the "profession almost unanimously condemns the action of the American Medical Association with reference to the International Congress, and furnishes a list of eighteen journals (and says it is a correct list) with such sentiments, in support of the assertion. There being about two hundred journals published in the United States, this list is only about nine per cent., and yet they express the "unanimous" voice of the profession !

Second Fiddler says "only four 'organs' support the Association—that of the Association, that of the treasurer (?), and those of Daniel and Shoemaker."

We have sixty-three exchanges. A review of them gives the following results :

The *Record's* list as opposing the action of the Association: *New York Medical Record*, *New York Medical Journal*, *Boston Medical Journal*, *Chicago Journal and Examiner*, *Maryland*

*Medical Journal, Medical Age, Medical Times, Louisville Medical News, Atlanta Medical and Surgical Journal, Virginia Medical Monthly, Indiana Medical Journal, Pacific Medical and Surgical Journal, American Practitioner, New Orleans Medical and Surgical Journal, Cincinnati Medical Journal, Columbus Medical Journal, Medical News, Kansas Index.*

Eighteen journals, representing fourteen States—seven Northern and Northwestern, and seven Southern and Southwestern.

[We will throw in the *N. C. Medical Journal* for luck.—ED.]

Our list, endorsing said action: *Gaillard's Medical Journal, The N. Y. Sanitarian, New England Medical Monthly, Baltimore Medical Chronicle, Mississippi Valley Medical Monthly, Detroit Lancet, Louisville Medical Herald, Southern Practitioner, St. Joseph Medical Herald, Journal American Medical Association, Medical Bulletin, College and Clinic Record,* DANIEL'S TEXAS MEDICAL JOURNAL.

Thirteen Journals, representing ten States, five northern and northwestern and five southern and southwestern—equally divided,so it cannot justly be charged as a " southern conspiracy.

List of neuter Journals: *St. Louis Medical and Surgical Journal, Ft. Wayne Medical Journal, Braithwaite's Retrospect, Eastern (Mass.) Medical Journal, Georgia Eclectic Journal, Independent Practitioner, Iowa Medical Examiner, Medical Era, Sanitary News, Medical Abstract, Southern Clinic, Courier of Medicine, Weekly Medical Review, American Medical Journal, California Medical and Surgical Journal, Denver Medical Times, Piffard's Journal, C. D. New York Anylist, New York Analect, Buffalo Medical and Surgical Journal, Lancet and Clinic, Western Medical Reporter, Brief, Archives of Pediatrics, Medical World, North Western Lancet, Polyclinic, Therapeutic Gazette, Leonard's Illustrated, Sanitary Monitor, Medical Times, Texas Courier Record.*

Thirty-two Journals representing fifteen States, ten northern and five southern,—neuter in the controversy.

It will be seen that of the sixty-three journals on our list, eighteen, or 28.5 per cent, representing fourteen States—seven Northern and seven Southern—oppose the Association. Thirteen, or 23 per cent, representing five northern and five Southern States, actively indorse the Association and denounce the

Obstructionists; while thirty-two, or 50.7 per cent, in five Southern and ten Northern States, are non-committal on the subject, and therefore *do not* "condemn the action of the Association." These latter, added to the twelve which endorse the Association, give a total of forty-five journals, or 71.5 per cent, representing twenty-five States—fifteen Northern and ten Southern—which we can cite as *not* "condemning the Association," as charged by the *Record*.

Comment is unnecessary. Ignorance of the sentiments of forty-five out of sixty-three medical journals, including two leading New York journals, is h-a-r-d-l-y probable; yet common honesty demands that these (mis)leading journals (not "organs," but fiddles, we'll call them) must plead ignorance, or stand convicted before the medical world of *deliberate wilful misrepresentation for the purpose of deceiving!*

